# Implementation of a surveillance and monitoring system for chronic disease: A scoping review: Running title: Chronic disease surveillance and monitoring system

**DOI:** 10.3934/publichealth.2026036

**Published:** 2026-06-24

**Authors:** Yejin Kim, Hyung-Seop Sim, Bomgyeol Kim, Vasuki Rajaguru, Tae Hyun Kim

**Affiliations:** 1 Graduate School of Cancer Science and Policy, National Cancer Center, Goyang 10408, Republic of Korea; 2 Department of Public Health, Graduate School, Yonsei University, Seoul 03722, Republic of Korea; 3 Mo-Im Kim Nursing Research Institute, College of Nursing, Yonsei University, Seoul 03722, Republic of Korea; 4 Department of Healthcare Management, Graduate School of Public Health, Yonsei University, Seoul 03722, Republic of Korea; 5 Department of Biohealth Industry, Graduate School of Transdisciplinary Health Sciences, Yonsei University, Seoul 03722, Republic of Korea

**Keywords:** chronic diseases, public health surveillance systems, surveillance evaluation, surveillance indicators, surveillance system monitoring

## Abstract

**Background:**

Numerous national-based indicators aid in designing population-centered chronic disease surveillance. However, there is little consensus on the key indicators and goals to develop a chronic disease surveillance analysis system.

**Objective:**

This study aimed to develop evidence-based indicators to measure and improve the health outcomes of chronic disease surveillance systems in Korea.

**Methods:**

A scoping review was focused on peer-reviewed literature from 2012 to 2022 using PubMed, Medline, Embase, PsychINFO, CINHAL, Wiley Online, Scopus, and Cochrane Library. Three reviewers evaluated and selected the articles in accordance with comprehensive inclusion and exclusion criteria. Data were synthesized using median descriptive analyses, prioritization, and agreement. There was a consensus meeting to discuss the recommendations, and the findings were thematically and descriptively analyzed.

**Results:**

Forty-eight articles were finalized and most of them were published in 2019 (18.8%). The studies on chronic disease-based surveillance systems and related data sources were primarily conducted in Canada (58.3%). The findings were prioritized by prevalence rate, risk factors, data linkage, complex disease, and updated indicators in a current system. The key themes of focus were mortality, survival management, diagnosis, treatment, and healthcare systems.

**Conclusion:**

Our preliminary measurement methods need validation through follow-up projects. Chronic disease surveillance will improve public health resource allocation and monitoring in real time. Public health and healthcare systems could be enhanced through timely assessments of population health at the local and regional levels. In Korea, experts will validate the chronic disease-based surveillance system's development.

## 1. Introduction

Chronic diseases, often known as noncommunicable diseases (NCDs), are major causes of death and have emerged as one of the primary challenges to sustainable human development in the 21st century [1]. The World Health Organization states that approximately 40 million individuals die annually from chronic diseases, accounting for 70% of all deaths [2]. In Korea, life expectancy has increased by approximately 21 years, from 62.3 in 1970 to 83.6 in 2021 [3]. Korea may become a superaged society by 2025, with people aged 65 or older constituting 20% or more of the overall population at an unprecedented rate [4]–[6] and 70% of disease burdens and 80% of fatalities occurring due to chronic diseases [3]. As a direct consequence, there is a strong possibility that the prevalence of chronic diseases may increase in the near future.

The World Health Assembly recognized “surveillance to track and monitor the major risk factors and the strengthening of technical and institutional surveillance capacities at the national level as a top priority for overcoming chronic diseases” [1],[5]–[8] in its first global strategy for the prevention and control of NCDs, which was adopted in 2000 [7]. The three main elements of the approach were health promotion to reduce these risk factors, surveillance to identify and track the main risk variables, and efficient management to increase access to healthcare [9],[10]. Chronic disease preventive services were disrupted by the coronavirus disease of 2019 (COVID-19); public health outreach and monitoring programs were replaced with pandemic control initiatives, making the fight against chronic diseases even more difficult [11].

Chronic disease surveillance is a scientifically validated aspect of public health, with the purpose of collecting, analyzing, and sharing data in a systematic manner so that interventions can be targeted and tracked to reduce disease mortality and morbidity [10]–[12]. In many low- and middle-income countries, surveillance and monitoring are regarded as inadequate [12]–[14]. Nevertheless, they considerably vary and are necessary to deliver population-based public health information and developing policies for NCDs prevention programs [8]–[10],[15].

The development of surveillance-based monitoring systems is essential, as they serve as a source of data to evaluate global and regional targets and as evidence for policymaking [16]. With these objectives, the Korean government initiated the Chronic Illness Care System program in 2012, thereby recognizing the importance of providing appropriate disease management to enhance the treatment quality and control costs [17].

In Korea, there are numerous surveys and registries on many chronic diseases, including cancer, diabetes, chronic obstructive pulmonary disease (COPD), stroke, and hypertension, which provide vital national epidemiological data on NCDs [18],[19]. However, information regarding NCDs has been collected by self-reported household surveys; monitoring data can only be obtained from a limited number of surveillance locations, and the quality varies by region. Thus, this study focused on the development of surveillance and monitoring systems to enable the gathering, analysis, and dissemination of NCDs data at the national level to improve public health policy [20].

The purpose of this study was to identify and compare national and worldwide guidelines to develop a set of evidence-based indicators and to measure the health outcomes of the chronic disease surveillance system (CDSS). Furthermore, plans of action and strategies for the establishment of Korea's chronic disease surveillance and monitoring analysis system in Korea should be developed.

## 2. Methods

This scoping review was conducted using the six stages developed by Arksey and O'malley [21] with recent advancements by Levac et al. [22], in accordance with the Preferred Reporting Items for Systematic Reviews and Meta-Analyses (PRISMA) statement and scoping review checklist (Supplementary Table S1).

### 2.1. Research question

The following research question was established by the researchers to accomplish the purpose of the study: What are the chronic disease surveillance-based indicators that can be implemented to measure the surveillance and monitoring system of chronic diseases?

### 2.2. Selection of relevant studies

The primary research and literature search focused on published scientific articles and existing international guidelines. The first draft list of surveillance-related indicators was compiled using a predetermined search strategy (Supplementary Table S2) and systematic searches of the electronic databases PubMed, CINAHL, Cochrane Library, and Ovid-EMBASE with search terms such as Chronic diseases*, NCD*, noncommunicable, muliti*, “multimorbidity”, prevent*, “public health”, “population health”, AND “Surveillance”, system*, monito*, “monitoring”, approach*, strateg*, determinant*, multidisciplin*, network*, organization*, multilevel, resource*, system* AND “indicators”, index* from the Medical Subject Headings, and the search period was limited to January 2012–December 2022 since chronic disease databases have become more widely used during the previous ten years. The eligibility criteria are presented in Table 1.

**Table 1.** Eligibility criteria for relevant article selection.

Content	Inclusion criteria	Exclusion criteria
Article types	All the original peer-reviewed research article	Review, case studies, conferences papers, commentary, editorial letter, thesis, textbooks, protocols
Language	Studies that were published in English language with Full text only	Article published other than English
Period	Recent 10 years 1, January 2012 to 31st December 2022	Studies published before 2012
Design	Studies focused on any kind of study designs	
Population	Publications from any country of origin or public and private organizations focused the indicators for the chronic disease surveillance system	Other than chronic disease surveillance system
Interventions	Studies that focused on data based driven development or implementation of chronic disease indicators that estimated and reported prevalence to surveillance system monitoring	Articles that were not related to chronic diseases' surveillance indicators

### 2.3. Data extraction and synthesis

We used the Research Information Systems format to export our data from online databases and imported them into EndNote (Ver.20, Clarivate, USA). Titles and abstracts were reviewed for eligibility, and duplicates were removed. Two researchers (Y.K. and V.R.) worked independently to choose the articles and retrieve the data. The screening process was repeated by three researchers (Y.K., B.K., and T.H.K.) to ensure reproducibility and to verify the outcomes. The data were extracted and the selected articles were organized. Three researchers independently examined the information obtained from the literature. When discrepancies arose, the researchers discussed them and reached a consensus to resolve them. An Excel spreadsheet was used to summarize the results.

The extracted data were collated from the reviewed articles and summarized according to the study characteristics, including the author(s), publication year, country, purpose, research design, setting, target population, and methods of data collection, with the number of indicators and their characteristics. The findings were presented as a narrative synthesis and reported based on the heterogeneous nature of the reviewed articles. The final report was retrieved and sorted into five themes that were based on the frequently applied indicators for NCDs. Since this study will be continued for future expert opinion, a quality assessment was not applicable.

## 3. Results

### 3.1. Literature search

The database yielded a total of 5939 articles, and 7 articles were obtained by manual search. After duplicates were eliminated, 2514 articles remained, of which 910 were chosen for screening. The titles and abstracts of the recovered documents were used to determine their eligibility. The final data synthesis included 48 articles (Figure 1).

**Figure 1.**
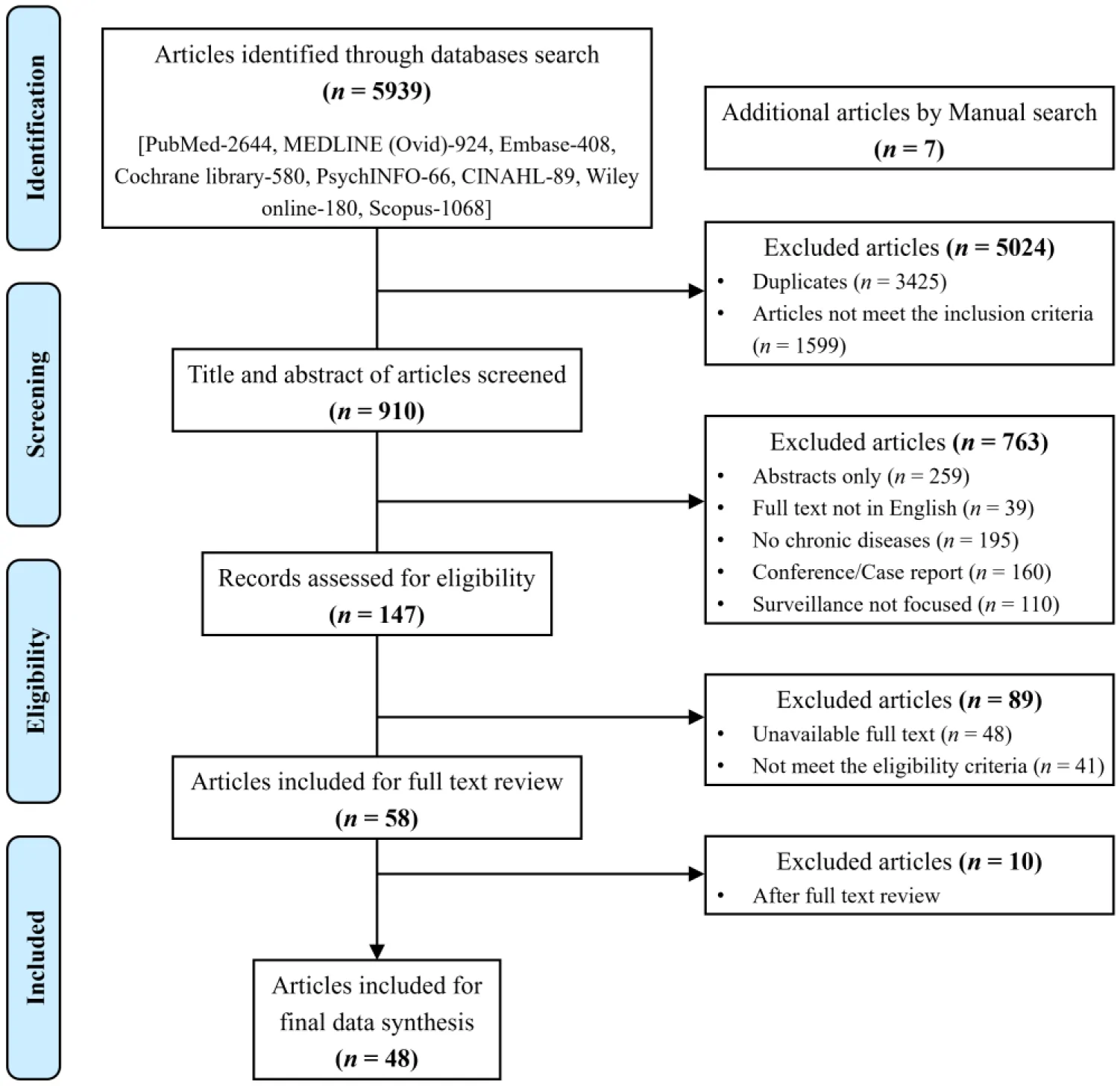
Flow chart for the article selection process.

### 3.2. Characteristics of the articles

Most articles were published after 2018 from various countries, including Canada (54.2%) [23]–[48], the United States of America (USA) (31.3%) [49]–[63], and China (8.3%) [64]–[67]. In terms of study designs, thirty studies used population-based cohorts (58.3%) [23]–[25],[28],[30],[31],[35]–[42],[44],[45],[47],[48],[51],[52],[54]–[60],[65],[68], thirteen studies used cross-sectional methods (37.5%) [27],[32]–[34],[46],[49],[53],[64],[66],[67],[69],[70], two studies used mixed methods (4.2%) [26],[61], one study used descriptive methods [29], and twenty-one studies focused on more than three chronic diseases [28],[29],[35],[37],[45]–[47],[52]–[56],[58],[60],[62],[63],[66]–[68],[70]. The details of the selected articles and their characteristics are presented in Supplementary Table S3 and Figure S1.

### 3.3. Data sources and surveillance systems

The most commonly used CDSSs and data sources are summarized in Table 2. Overall, surveillance relied on a limited set of dominant data sources, primarily national surveys, administrative health data, and electronic health records (EHRs). These systems predominantly focused on cardiometabolic, endocrine/metabolic, and respiratory diseases, while multimorbidity and less common chronic conditions were less frequently addressed.

**Table 2.** List of data resources and surveillance systems from the selected articles.

Country	Data source/Surveillance system	Category of chronic diseases*	References
Canada	Quebec Integrated Chronic Disease Surveillance System	Cardiometabolic, renal, and mental health disorders	[23],[25],[28]–[31],[35],[51]
	Canadian Community Health Survey	Cardiometabolic, mental health, and respiratory diseases	[26],[32]–[34],[45]
	Canadian Health Measures Survey	Cardiometabolic diseases	[26],[33]
	Canadian Primary Care Sentinel Surveillance Network	Cardiometabolic, and mental health disorders	[26],[32],[33],[36]
	Electronic Medical Records/Electronic Health Record	Cardiometabolic, endocrine/metabolic, renal, respiratory, mental health, and cancer	[45],[54],[56],[59]–[61],[69]
	Canadian Chronic Disease Surveillance System	Cardiometabolic, endocrine/metabolic, respiratory, and mental health disorders	[26],[32],[33],[36],[37],[41],[43],[44]
	Health Insurance Registry (National/Regional Health insurance)	Cardiometabolic disease, and multimorbidity	[31], [40]
	- National Population Health Survey - Composite International Diagnostic Interview Short Form	Mental health disorders	[34]
	First Nations Community Based Screening to Improve Kidney Health and Prevent Dialysis	Renal diseases	[38]
	Public Health Agency of Canada	Multimorbidity	[40]
	Manitoba Centre for Health Policy	Endocrine/metabolic diseases	[42]
	Alberta Health Administrative Data	Cardiometabolic, endocrine/metabolic, respiratory, and musculoskeletal disorders	[47]
USA	- Electronic Health Record Public Health Information Exchange - University of Wisconsin - Primary Care Clinic Data	Respiratory diseases	[49]
	Behavioral Risk Factor Surveillance System or Wisconsin Behavioral Risk Factor Surveillance System	Endocrine/metabolic, and respiratory diseases	[49],[50]
	- New York City Health and Nutrition Examination Survey - Community Health Survey	Cardiometabolic, endocrine/metabolic, and respiratory diseases	[52],[55],[57],[60]
	Macroscope Electronic Health Record	Cardiometabolic, and endocrine/metabolic diseases	[60]
	Cancer Registry Data	Cancer	[61]
	Colorado Health Observation Regional Data Service	Cardiometabolic, endocrine/metabolic, respiratory, mental health, musculoskeletal diseases, and cancer	[62],[63]
China	Hospital Quality Monitoring System	Renal diseases	[64]
	Zhejiang Chronic Disease Surveillance Information and Management System	Injury related chronic diseases	[65]
	Chinese Center for Disease Control and Prevention	Endocrine/metabolic diseases	[66]
	National Health Services Survey	Cardiometabolic, endocrine/metabolic, respiratory, and other chronic conditions	[67]
Australia	General Practice Clinical Systems	Cardiometabolic, endocrine/metabolic, respiratory, mental health, musculoskeletal diseases, and cancer	[68]
	- Progessi Delle Aziende Sanitane per la Salute in Italia - South Australian monitoring and surveillance system	Cardiometabolic and endocrine/metabolic diseases	[70]
Portugal	Electronic Health Record Administration data	Endocrine/metabolic diseases	[69]

Note: USA = United States of America. * These reflect the chronic diseases addressed in the studies included in this scoping review.

In Canada, surveillance was largely anchored in national platforms, including the Canadian Community Health Survey [26],[32]–[34],[45], the Canadian Primary Care Sentinel Surveillance Network [27],[39],[46], and the Canadian Chronic Diseases Surveillance System [26],[32],[33],[36],[37],[41],[43],[44]. These systems were complemented by provincial administrative databases such as the Quebec Integrated Chronic Diseases Surveillance System [23],[25],[28]–[31],[35],[48]. Additional data sources included health insurance registries [31],[36],[40] and the Manitoba Centre for Health Policy [42], and EHR-based data sources were used in several studies [45],[47],[56],[69]. Across these systems, the studies covered a broad range of chronic disease categories, including cardiometabolic, mental health, endocrine/metabolic, respiratory, and renal conditions.

In the USA, surveillance systems commonly included national surveys such as the Community Health Survey [52],[55],[57] and the Behavioral Risk Factor Surveillance System [49],[50]. Local and regional systems were also used, including the Colorado Health Observation Regional Data Service [62],[63] and the New York City Health and Nutrition Examination Survey [55]–[57],[60]. Across these systems, the studies primarily focused on monitoring cardiometabolic [52],[60],[62],[63], endocrine/metabolic [50],[60],[62],[63], and respiratory diseases [49],[52],[55],[57],[60],[62],[63]. Some systems also captured additional disease categories, including cancer, mental health, and musculoskeletal disorders [61]–[63].

In China, surveillance systems were based on nationally coordinated or institution-based databases, including the Hospital Quality Monitoring System [64], the Zhejiang Chronic Diseases Surveillance Information and Management System [65], the Chinese Center for Disease Control [66], and the National Health Services Survey [67]. These systems tended to focus on specific disease categories (e.g., chronic kidney disease, and injury related chronic diseases), although the national survey systems covered multiple conditions.

**Figure 2.**
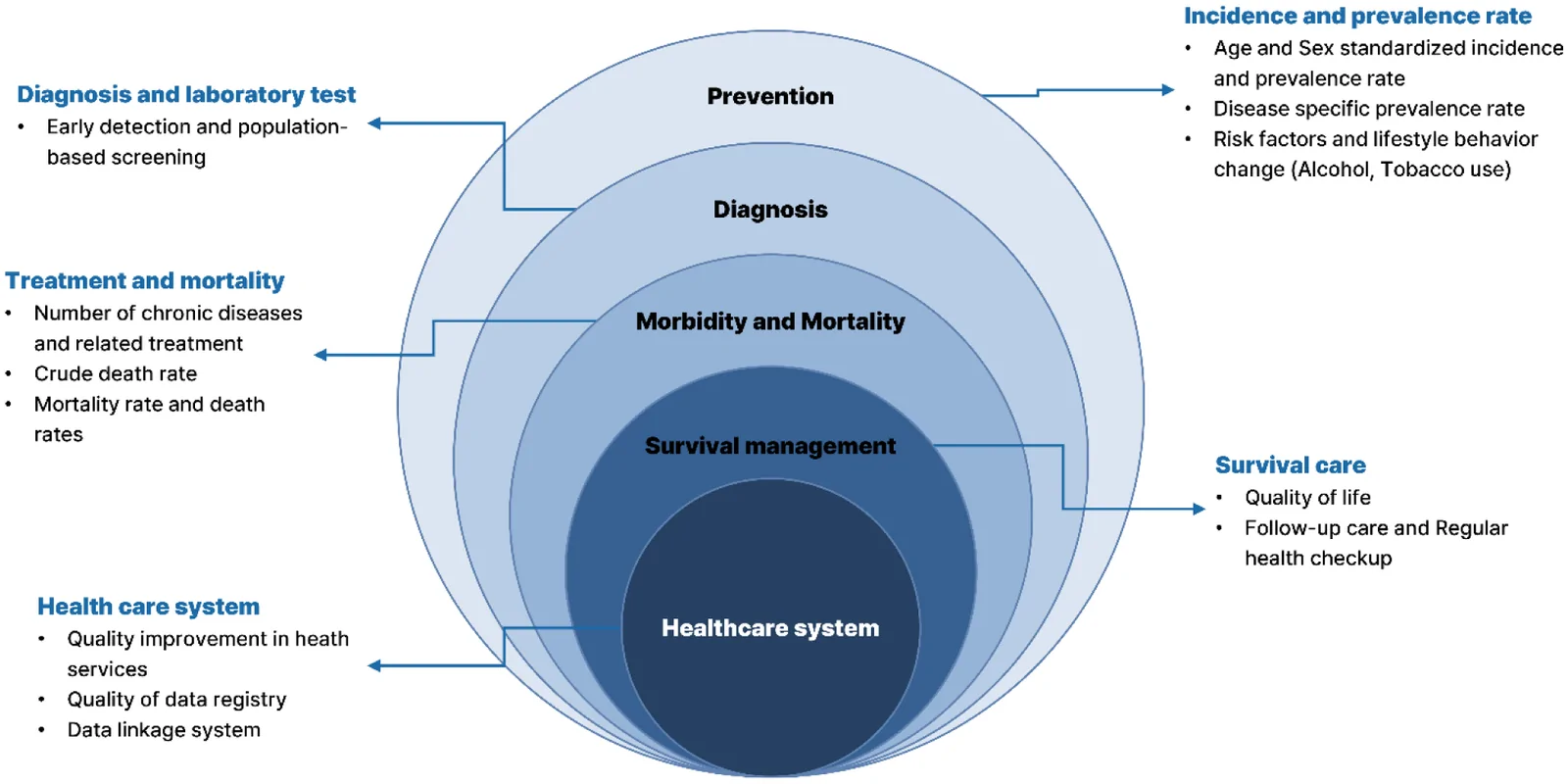
Commonly focused indicators in the selected articles related to the chronic disease surveillance system.

### 3.4. Chronic disease surveillance-related indicators

This review obtained key indicators relevant to the CDSS from the primary and secondary outcomes of the selected articles. Most of the chronic diseases and relevant surveillance systems were categorized by roadmap according to priority: prevalence rate, risk factors, data linkage, complex disease, and update of the existing system (Supplementary Figure S2). Figure 2 shows the five domains of chronic diseases that have been widely investigated, including prevention, diagnosis, treatment, survival care, and the healthcare system. The concentric structure represents the continuum from prevention to outcomes and system-level support. Each domain includes the following key indicators: prevention (incidence and risk factors), diagnosis (screening), morbidity and mortality (disease burden), survival care (follow-up and quality of life), and the healthcare system (data and service infrastructure).

**Table 3.** Common indicators retrieved from the selected articles on chronic diseases and the surveillance system.

Indicators	Content	References
Prevention	Prevalence	Age and sex standardized prevalence rate (National/Regional and disease based)	[25]–[27],[30]–[33],[49],[50],[61],[65],[67],[68]
	Incidence	Age and sex standardized incidence rate (National/Regional and disease based)	[24],[31],[35],[41]–[43],[47]
	Risk factors	- Risk factors influencing the onset and progression of the disease - Lifestyle modification behavior by specific population	[26],[34],[39],[70]
Diagnosis	Identification of complex chronic diseases	Detection of a complicated chronic illness and morbidity (two or more chronic diseases)	[23],[27],[36],[38],[40],[64]
Treatment	Morbidity and mortality	- Determinants of patients of more than two chronic diseases by region or national - Healthcare utilization (e.g., frequency of emergency room visits, hospitalization, and outpatient health services) - Percentage and cause of death or death rate (all cause or specific disease) by annual	[28],[29],[35],[36],[40],[55] [30],[31],[33],[35],[38],[39],[49],[53],[56]
Survival management	Quality of life, Life expectancy, Follow up and welfare service	- Estimation of the quality of life and life expectancy by patients with CKD and mental health disorders - Welfare services for parents with injury related disability (CKD and mental health disorders) - Outcomes of education/awareness	[28],[34],[38],[39],[44],[49],[57]–[59],[65]
Healthcare system	Data linkage	Linking each disease's surveillance system with data from other surveillance systems to monitor NCDs	[50],[65],[67],[68] [23]–[34],[38],[49],[61],[65]
	Update	- Varying the frequency of updates according to disease epidemiology, intervention strategies, and available local resources - Preferably conducting data collection at least once every five years, or more frequently when feasible	[28],[34],[38],[49],[54],[57],[59]
	Additional indicators	Health care systems, health care quality and health policy goals	[32],[34],[35],[38],[41],[49],[54],[57],[59]

Note: CKD = chronic kidney disease; NCDs = noncommunicable diseases.

Table 3 presents key indicators for chronic disease surveillance across prevention, diagnosis, treatment, survival management, and healthcare systems. Prevention focuses on three sub-indicators: prevalence and incidence rates [24],[31],[35],[36],[41]–[43],[47],[62], risk factors, and lifestyle behaviors [26],[34],[39],[70]. Diagnosis emphasizes identifying multimorbidity, while treatment includes measures of morbidity, mortality, and healthcare utilization [28],[29],[35],[36],[40],[53],[55]. Survival management captures quality of life, life expectancy, follow-up care, and welfare services, particularly for conditions such as CKD and mental health disorders [28],[34],[38],[39],[44],[49],[57]–[59],[65]. Healthcare system indicators highlight data linkage, regular updates [28],[34],[38],[49],[54],[57],[59], and measures of healthcare quality and policy, thus reflecting a comprehensive framework to monitor NCDs [32],[34],[35],[38],[41],[49],[54],[57],[59].

## 4. Discussion

This scoping review aimed to identify relevant articles on chronic disease surveillance and monitoring from 2012 to 2022 using electronic databases. A total of forty-eight articles were finalized for data synthesis by the research team. Most of the publications that were chosen were published after 2018; therefore, it appears that the evidence is recent and up to date [23],[25],[27]–[30],[35],[38]–[43],[48],[50],[53],[54],[58],[59],[64],[66],[67],[70]. The development of CDSSs is related to significant improvements in monitoring indicators, but not always to improvements in intermediate or long-term outcomes. Hence, there may be an opportunity and a need to develop more robust chronic disease surveillance models in the population-based setting.

Most of the studies were conducted using data from the CDSS in Canada, and the following studies were related to the design of confirming the data quality of the existing surveillance system [31],[42],[44],[45],[52],[54]–[57],[60],[61],[65],[69],[70]. Public health surveillance systems have been used to monitor and forecast a wide range of health determinants (such as risk behaviors, health care services, and socioeconomic factors) and outcomes relevant to chronic diseases. Historically, these systems have focused on infectious diseases. Based on our findings, implementing EHR-based surveillance systems remained challenging. Issues such as limited interoperability, differences in digital infrastructure, and gaps in the workforce capacity affected system performance. In addition, variations in data standards reduced the comparability. These findings highlight the need for better integration, standardization, and workforce development [71],[72]. The utilization of EHR-based chronic disease surveillance is still in progress. The most typical method of validation is to compare estimates from EHR-derived data to those from conventional nationwide surveillance surveys [45],[49],[54],[56],[59]–[61],[69]. This strategy has yielded consistent outcomes across reviewed studies.

There were thirteen surveillance systems identified from the articles published in Canada. Most of them monitor the prevalence and risk factors in terms of prevention indicators by focusing on specific diseases, such as hypertension [26],[33], chronic kidney disease [23],[38], substance-related disorders [30], and mental health [29], which have been utilized to monitor the NCDs prevention and control system. Prevention-based indices were focused on using national, regional, and combined surveillance system databases [62],[63]. Most of the articles focused on the prevalence rate of multimorbidity using combined databases [26],[32]–[34],[45]. The regional-based surveillance system reported a prevalence rate of mental health disorders such as the spectrum of substance-related disorders [30]. It was found that cardio- and cerebrovascular diseases were the most common among other diseases and that their prevalence rates have increased [28],[29],[31],[35],[37],[40],[45],[47],[54],[58],[67],[68]. Therefore, we decided to initiate cardiovascular-related surveillance monitoring, including monitoring for cerebrovascular disease related hypertension and diabetes as a first priority.

Seven studies used technology, population-based screening, and blood sample collection to diagnose NCDs [23],[27],[36],[38],[40],[50],[64], such as multimorbidity, chronic kidney disease, and diabetes (HbA1C). A system that enables the performance of surveillance may consist of the following components: laboratory diagnostics to identify or confirm health conditions; information technologies to assist with data collection, analysis, and transfer; worker education and training; and laws, regulations, and procedures that support surveillance. For multimorbidity surveillance system monitoring, the Canadian Primary Care Sentinel Surveillance Network diagnostic algorithms notably demonstrated outstanding sensitivity and specificity [46],[62].

Each data source used for chronic disease surveillance has distinct strengths and limitations. Health surveys, which are typically national or community-based, population-representative, and regularly conducted, provide a wide range of information on health behaviors, demographics, and socioeconomic factors, but they rely on self-reported data, which may introduce recall bias and limit the assessment of comorbidities [43],[66],[67]. EHRs offer the potential for real-time and standardized clinical data, although their integration into large-scale surveillance systems remains limited [55]. The use of clinical data and health insurance data may be advantageous for the creation of an NCD-based surveillance system. The clinician's effort and the duration of the patient's visit were both diminished due to the reduction in duplicate entries and recurrent demands [62],[63],[68], but these types of data may be limited by incomplete case ascertainment and challenges in accurately capturing the disease severity and burden [43],[45]. Ghosh et al. [68] demonstrated that it is possible to estimate the prevalence of more than five chronic diseases by region using clinical data from Australia's general clinical surveillance system. Previous studies have demonstrated that accurate prevalence and incidence rates for cardiovascular disease may be obtained using administrative and health insurance data, and case definitions for multimorbidity can be used [31],[40].

In a sentinel system, systematic reporting unit classification is necessary to ensure data accuracy. Data registries are very helpful to obtain clinical data for all patients with specific or chronic diseases within a particular region over time via population-based surveillance systems. Studies that have estimated the prevalence of NCDs only using the EHR administrative data or connected with additional data have shown potential for utilization in chronic illness public health surveillance [69]. This would be essential for patient care and treatment, especially to determine the regional prevalence without population-level patient monitoring systems. However, establishing electronic medical record (EMR) systems requires proper electronic infrastructure, especially for EMR- or EHR- based systems. Primary care facilities have the necessary infrastructure to receive and process EMR-based reports.

Our findings showed that the collection of more precise data is made possible by combining population-based strategies with facility-based surveillance systems. Periodic surveys provide previously unavailable data on subclinical symptoms, risk factors, illness knowledge, socioeconomic information, health system accessibility, and health-seeking habits. The combination of national, regional, and clinical data has also been used in a number of countries [26],[27],[31],[32],[49],[52]–[55],[60]. It may be challenging to connect information from recurring population-based surveys with regular facility-based monitoring systems in the absence of a unique identification number (ID).

The predominance of Canadian studies in this review likely reflected the strength and long-standing development of Canada's chronic disease surveillance system, particularly the Canadian CDSS. This system enabled the integration of administrative health data across provinces, thus supporting consistent, large-scale monitoring of chronic disease indicators over time. In addition, Canada had well-established data linkage mechanisms and standardized reporting frameworks, which facilitated the generation of high-quality, publishable research. These structural and methodological advantages likely contributed to the higher visibility of Canadian studies in the peer-reviewed literature [73],[74].

In contrast, other countries appeared less represented not because of weaker systems, but due to differences in how surveillance information was documented and disseminated. For example, countries such as the United Kingdom (UK) and other centralized health systems often reported surveillance activities through national guidelines, technical reports, or government publications rather than journal articles. Furthermore, variations in healthcare systems, data accessibility, and digital infrastructure limited cross-country comparability. These differences highlighted the importance of interpreting findings within their specific context and carefully adapting internationally derived indicators to the Korean setting. Additionally, they underscored the need to integrate both global evidence and locally relevant data sources in future surveillance system developments [71],[75].

Over time, surveillance indicators shifted from survey-based measures to more integrated, data-driven approaches. Earlier studies relied on surveys and registries, while studies after 2018 increasingly incorporated EHRs and administrative data. This trend reflects the growing emphasis on timely, comprehensive data for public health decision-making [71],[72]. In the Korean context of surveillance system monitoring, data linkage is technically feasible due to the existence of a universal national health insurance system and unique personal ID. However, such linkage is currently limited to claim data. In addition, integrating data across different sources may raise important concerns regarding data privacy, security, and governance, as well as technical challenges related to harmonizing heterogeneous databases. While many existing datasets in Korea provide de-identified ID to researchers, linkage through unique personal ID may increase the risk of re-identification as more comprehensive individual-level information is aggregated. As a result, trade-offs may be required between data utility and privacy protection, potentially necessitating the exclusion or modification of certain variables during the data integration process. Furthermore, differences in data collection periods and update cycles across data sources should also be considered, as temporal inconsistencies may affect the validity and interpretability of integrated data.

Prioritizing research on NCDs-related indicators and applying measures and results that are accurate and consistent over time are necessary before creating common objectives can be undertaken. Previous studies and literature reviews on the surveillance of chronic diseases have not shown any consistency in terms of the studies priorities or key results. Within the confines of offline or online engagement, achieving agreed-upon goals through deductive consensus approaches is a transparent and verifiable process.

### 4.1. Limitations

This study has several limitations. First, the search was restricted to English-language publications, which may have excluded relevant studies, including those from Korea, and contributed to the predominance of Western-based evidence, thus limiting contextual generalizability. Second, countries with advanced surveillance infrastructures, such as the UK and other centralized health systems, may be underrepresented, as key frameworks are frequently reported in national policy documents or technical reports rather than peer-reviewed literature. Third, the focus on surveillance indicators excluded measures of intervention processes, implementation fidelity, and effectiveness, thereby limiting a comprehensive assessment of public health impact and system performance. Fourth, although large national datasets (e.g., National Health and Nutrition Examination Survey and other administrative or EHR-based systems) are highly relevant for surveillance, few eligible studies met the inclusion criteria, thus reflecting variability in the reporting standards and limiting comparability across settings. Finally, the emphasis on major NCDs may have overlooked ageing-related conditions, multimorbidity complexity, and emerging chronic disease patterns, which are increasingly important in rapidly ageing populations such as Korea.

## 5. Conclusion

This research revealed updated databases that enable the sharing of chronic disease surveillance data on various platforms. By linking the various sources stated earlier, a reasonably complete personal health file can be obtained at the conclusion of the process. This enables the development of risk prediction models and the incorporation of follow-up data, mortality data, and EHR data into various NCDs cohorts. The proposed indicators will require further validation in future studies. This will include assessing their feasibility, reliability, data availability, and relevance for policy use. Follow-up work, including expert consultation and pilot testing with national health data, will help ensure that these indicators are practical and applicable in real-world settings. It is possible to address several categories of risk factors and carry out treatments based on evidence. These research findings might be helpful in the establishment of evidence-based policy.

## Use of AI tools declaration

During the preparation of this work, the authors used ChatGPT solely to improve readability and revise the English in some parts of the work. After using this tool, the authors have reviewed and edited the content as needed and take full responsibility for the content of the publication.


