# Supplementary material for: Implementation of a surveillance and monitoring system for chronic disease: A scoping review: Running title: Chronic disease surveillance and monitoring system
Source: AIMS Public Health. 2026 Jun 24;13(3):671–88. doi: 10.3934/publichealth.2026036 (PMC13684081; doi:10.3934/publichealth.2026036)
Supplement: Supplementary file 1 [file publichealth-13-03-036-s001.pdf]

Public Health

AIMS Public Health, 13(3): 671–688.
DOI: 10.3934/publichealth.2026036
Received: 31 December 2025
Revised: 04 April 2026
Accepted: 23 April 2026
Published: 24 June 2026

http://www.aimspress.com/journal/aimsph

Review

Implementation of a surveillance and monitoring system for chronic disease: A scoping review

Running title: Chronic disease surveillance and monitoring system

Yejin Kim[1], Hyung-Seop Sim[2], Bomgyeol Kim[3], Vasuki Rajaguru[4] and Tae Hyun Kim[5,*]

[1] Graduate School of Cancer Science and Policy, National Cancer Center, Goyang 10408, Republic of Korea
[2] Department of Public Health, Graduate School, Yonsei University, Seoul 03722, Republic of Korea
[3] Mo-Im Kim Nursing Research Institute, College of Nursing, Yonsei University, Seoul 03722, Republic of Korea
[4] Department of Healthcare Management, Graduate School of Public Health, Yonsei University, Seoul 03722, Republic of Korea
[5] Department of Biohealth Industry, Graduate School of Transdisciplinary Health Sciences, Yonsei University, Seoul 03722, Republic of Korea

* **Correspondence:** Email: thkim@yuhs.ac; Tel: +8222281521.

Table S1. Preferred reporting items for systematic reviews and meta-analyses extension for scoping reviews (PRISMA-ScR) checklist.

SECTION	ITEM	PRISMA-ScR CHECKLIST ITEM	REPORTED ON PAGE #
TITLE			
Title	1	Identify the report as a scoping review.	
ABSTRACT			
Structured summary	2	Provide a structured summary that includes (as applicable) background, objectives, eligibility criteria, sources of evidence, charting methods, results, and conclusions that relate to the review questions and objectives.	1–2
INTRODUCTION			
Rationale	3	Describe the rationale for the review in the context of what is already known. Explain why the review questions/objectives lend themselves to a scoping review approach.	2–3
Objectives	4	Provide an explicit statement of the questions and objectives being addressed with reference to their key elements (e.g., population or participants, concepts, and context) or other relevant key elements used to conceptualize the review questions and/or objectives.	3
METHODS			
Protocol and registration	5	Indicate whether a review protocol exists; state if and where it can be accessed (e.g., a Web address); and if available, provide registration information, including the registration number.	3
Eligibility criteria	6	Specify characteristics of the sources of evidence used as eligibility criteria (e.g., years considered, language, and publication status), and provide a rationale.	4
Information sources*	7	Describe all information sources in the search (e.g., databases with dates of coverage and contact with authors to identify additional sources), as well as the date the most recent search was executed.	3–4
Search	8	Present the full electronic search strategy for at least 1 database, including any limits used, such that it could be repeated.	3
Selection of sources of evidence†	9	State the process for selecting sources of evidence (i.e., screening and eligibility) included in the scoping review.	4
Data charting process‡	10	Describe the methods of charting data from the included sources of evidence (e.g., calibrated forms or forms that have been tested by the team before their use, and whether data charting was done independently or in duplicate) and any processes for obtaining and confirming data from investigators.	4
Data items	11	List and define all variables for which data were sought and any assumptions and simplifications made.	4
Critical appraisal of individual sources of evidence§	12	If done, provide a rationale for conducting a critical appraisal of included sources of evidence; describe the methods used and how this information was used in any data synthesis (if appropriate).	4
Synthesis of results	13	Describe the methods of handling and summarizing the data that were charted.	4

Continued on next page

SECTION	ITEM	PRISMA-ScR CHECKLIST ITEM	REPORTED ON PAGE #
RESULTS			
Selection of sources of evidence	14	Give numbers of sources of evidence screened, assessed for eligibility, and included in the review, with reasons for exclusions at each stage, ideally using a flow diagram.	4–5
Characteristics of sources of evidence	15	For each source of evidence, present characteristics for which data were charted and provide the citations.	5–6
Critical appraisal within sources of evidence	16	If done, present data on critical appraisal of included sources of evidence (see item 12).	6
Results of individual sources of evidence	17	For each included source of evidence, present the relevant data that were charted that relate to the review questions and objectives.	7–8
Synthesis of results	18	Summarize and/or present the charting results as they relate to the review questions and objectives.	8–9
DISCUSSION			
Summary of evidence	19	Summarize the main results (including an overview of concepts, themes, and types of evidence available), link to the review questions and objectives, and consider the relevance to key groups.	9–11
Limitations	20	Discuss the limitations of the scoping review process.	11
Conclusions	21	Provide a general interpretation of the results with respect to the review questions and objectives, as well as potential implications and/or next steps.	12
FUNDING			
Funding	22	Describe sources of funding for the included sources of evidence, as well as sources of funding for the scoping review. Describe the role of the funders of the scoping review.	12

Note: JBI = Joanna Briggs Institute; PRISMA-ScR = Preferred Reporting Items for Systematic reviews and Meta-Analyses extension for Scoping Reviews. * Where *sources of evidence* (see second footnote) are compiled from, such as bibliographic databases, social media platforms, and Web sites. † A more inclusive/heterogeneous term used to account for the different types of evidence or data sources (e.g., quantitative and/or qualitative research, expert opinion, and policy documents) that may be eligible in a scoping review as opposed to only studies. This is not to be confused with *information sources* (see first footnote). ‡ The frameworks by Arksey and O'Malley (6) and Levac and colleagues (7) and the JBI guidance (4, 5) refer to the process of data extraction in a scoping review as data charting. § The process of systematically examining research evidence to assess its validity, results, and relevance before using it to inform a decision. This term is used for items 12 and 19 instead of "risk of bias" (which is more applicable to systematic reviews of interventions) to include and acknowledge the various sources of evidence that may be used in a scoping review (e.g., quantitative and/or qualitative research, expert opinion, and policy document).

Table S2. Search terms.

Data bases	Search terms
PubMed / Science direct	
1	"Chronic disease*" [All Fields] OR "Non-communicable" OR "Non communicable diseases [MeSH Terms]" OR "NCDs" OR "Non-infectious Diseases" OR "comorbidity" OR chronic illness* OR noncommunicable (with a specific focus on high burden diseases: chronic respiratory diseases, cardiovascular diseases and diabetes)<br><br>CARDIOVASCULAR DISEASES (e.g.,'heart attacks and stroke'), CANCER, chronic respiratory diseases (e.g., 'CHRONIC OBSTRUCTIVE PULMONARY DISEASE' and 'ASTHMA') and 'DIABETES MELLITUS'.
2	"Public health" OR "Public health surveillance" OR "surveillance" OR "sentinel surveillance" OR "epidemiology" OR "population surveillance" OR "epidemiological monitoring
3	"Health information systems ("information systems", "hospital information systems", "health information management", "health information systems", "management information systems", "geographic information systems", "integrated advanced information management systems", "ambulatory care information systems", "information management", "automatic data processing", "electronic health records")
4	"Healthcare service" OR "Health assessment" OR "Risk management" OR "QOL" OR "Health survey" OR "Healthcare quality" OR "access" OR 'Diagnosis" OR "Treatment" OR "survival"
Remarks	['1' AND '2'] OR ['1' AND '2' AND '3'] OR ['1' AND '2' AND '3' AND '4']
EMBASE	
#1	('chronic disease'/exp OR 'chronic disease') ('non communicable disease'/exp OR 'non communicable disease') OR ('heart disease'/exp OR 'heart disease')<br>('Diabetes mellitus' /exp OR 'Diabetes' OR 'DM') OR ''chronic disease indicator'
#2	('surveillance system'/exp OR 'surveillance system') AND ('surveillance system'/exp OR 'surveillance system') OR ('information system'/exp OR 'information system')
#3	('Indicators'/exp OR 'Index') OR (Prevent*/exp OR 'prevention' OR promotion')
Remarks	#1 + #2 + #3
Cochrane library	
1	Chronic diseases*, NCD*, non-communicable, muliti*, "multimorbidity"
2	"Surveillance", system*, monito*, "monitoring", approach*, strateg*, determinant*, multidiscipline*, network*, organization*, multi-level, resource*, system*
3	prevent*, "public health", "population health", "indicators", index*
Remarks	[1 AND 2 AND 3] OR [1 AND 3] OR [1 AND 2 AND 3]
MEDLINE (Ovid)	
1	Chronic disease* OR (disease* OR condition* OR illness*) OR multimorbid OR multimorbidit* OR chronic medical.ab,ti.
2	Surveillance.ab,ti OR 'Monitoring syst*' OR Sentinel.ab,ti OR Syndromic Surveillance.ab,ti OR Sentinel Health.ab.ti
3	indicator.ab,ti. or managment.ab,ti. or monitoring.ab,ti. or parameter.ab,ti. or ((clinical or laboratory) adj3 (data or assess* or finding* or observ* or monitor* or examin* or check* or control*)). ab,ti. or ((data or information) adj6 (set or extract* or analys* or retriev* or yield* or gather*))
Remarks	1 AND 2 AND 3

All the search terms filtered by "title, abstract, keywords" and word variations have been searched in each term.

Table S3. Characteristics of the selected articles and their primary outcomes.

Ref	Author /Year/ Country	Design	Age (Years) /N	Chronic diseases	Data source	Surveillance measures / indicators	Attribute (results)	Primary outcome
23	Khelifi et al., 2022, Canada	Cohort	≥20<br>108 780	Chronic kidney disease (CKD)	QICDSS	Age and sex -standardized annual proportions of CKD<br>status and burden	Age-standardized annual Pre-dialysis proportions of males significantly higher than females	TRENDS and relationship between<br>AS and CKD
24	Al-Sakran et al., 2018, Canada	Cohort	NA<br>200 adults	Multiple sclerosis (MS)	Population-based health, administrative data	Age and sex -standardized MS incidence rate	Incidence of MS<br>↑ 313.6 per 100,000	Province-wide estimates of MS incidence
25	Leclerc J et al., 2020, Canada	Cohort	≥15<br>8.3 million	Mental health (depressive, bipolar and adjustment disorders)	QICDSS<br>(2000–2017)	Annual prevalence rate of mental health	(↓) Depressive disorders<br>(↓) bipolar disorders<br>(↑) adjustment disorders	Estimation for mental health care
26	Atwood et al., 2013, Canada	Cross sectional Cohort	20 to 79<br>4.9 million cases	HTN	CCDSS, CCHS, CHMS	Estimation of risk factors and Prevalence rate of HTN	(↑) Prevalence and related risk factors	Risk factors and prevalence of HTN
27	Bello AK et al., 2019, Canada	Cross sectional	≥18<br>559,745 adults	CKD	CPCSSN<br>EMRs	Estimation of the prevalence and severity of CKD	(↑) Prevalence of CKD (71.9 per 1000 individuals) prevalent among individuals with 3 or more other chronic diseases (281.7 per 1000)	Burden of moderate-to-advanced CKD and prevalence
28	Gaulin M et al., 2019, Canada	Cohort	≥18<br>5,316,832<br>Adults	Physical multimorbidity<br>Mental health	QICDSS<br>2012–2016	Estimation<br>on frequent visits to the emergency department (≥3 visits/yr.	↑ Mental disorders<br>↑ Physical conditions common<br>↑Higher ER visits and serious	Emergency<br>department visits for number of physical and menta disorders
29	Fleury MJ et al., 2019, Canada	Descriptive study	≥12<br>865,255 for 2014–2015;<br>817,395 for 2005–2006.	Mental health	QICDSS<br>2005–2006 and 2014–2015	Quality improvement, access, continuity, and appropriateness mental health services in relation to ER visits	↑ Frequency of ER use and LoS almost twice for PMI patients in 2005–2006 compared to 2014–2015	Quality of mental health services in relation to emergency room (ER) use to assess the impact
30	Huynh C et al., 2021, Canada	Cohort	≥12<br>NA	Spectrum of substance-related disorders (SRD)	QICDSS<br>2005–2006 and 2014–2016	Estimates prevalence and mortality rates of SRD	Annual prevalence rate was significantly higher in males<br>Alcohol-induced disorder<br>(2.1 per 1000)<br>Drug-induced disorder<br>(1.9 /1000)	age-adjusted prevalence rate and mortality of SRD

Continued on next page

Ref	Author /Year/ Country	Design	Age (Years) /N	Chronic diseases	Data source	Surveillance measures / indicators	Attribute (results)	Primary outcome
31	Blais C et al., 2015, Canada	Cohort	≥20 593,000	Coronary heart disease (CHD)	QICDSS Health insurance registry, vital statistics death database	Estimates the prevalence, incidence, and mortality	crude prevalence of CHD was 9.4% age-standardized prevalence increased 14%, Age-standardized incidence and mortality rates decreased by 46% and 26%	selection criterion allowing the application of case definitions for chronic disease surveillance
32	O'Donnell et al., 2015, Canada	Cross sectional	≥12 27 million	Mood and/or anxiety disorder	CCHS, CCDSS 2003 to 2009	Estimates the prevalence of mental health mood disorder	↑ Age-standardized mood and/or anxiety disorders ↑ Prevalence estimation in female	Prevalence rates compared across the CCHS and CCDSS
33	Padwal et al., 2016, Canada	Cross sectional	≥20 111,201	HTN	CCDSS, CCHSC CHMS	Estimation of crude and age-standardized self-reported prevalence awareness, and control	↑ Proportion of HTN prevalence and disease control, 68.1% ↓ Age-standardized mortality rates	update of the epidemiology of HTN
34	Steensma et al., 2016, Canada	Cross sectional	≥20 12,373	Mental health _Depression	NPHS, CCHS, CIDI-SF	Morbidity and mortality, Life expectancy	HRQOL values varied male and female; women aged 65 had higher depressive symptoms	HRQOL, HALE
35	Blais C et al., 2020, Canada	Cohort	≥20 473,305	Cardio and cerebro vascular diseases CHD), CVD, PAD	QICDSS 2015–2016	Estimation of prevalence and the all-cause crude mortality rate	Age-standardized prevalence increased relatively by 21.4% age standardized incidence and mortality rates showed relative decreases of 46.2% and 32.6%,	Trends in the burden of vascular diseases
36	Ellison J et al., 2016, Canada	Cohort	All age group 34,429,804	DM	CCDSS-2009 Health insurance registry, fee-for-service physician services file	Prevalence, Incidence of DM, All-cause mortality	CCDSS data source, researchers can make decisions about calculations of disease frequency	Calculate the differences between the CCDSS and Statistics
37	Feely A et al., 2017, Canada	Cohort	≥40 1 million Adult population	Cardiovascular disease, Respiratory disease, Mental illness, HTN, DM	CCDSS 2015	Estimates the multimorbidity prevalence and multimorbidity	There was a strong association between the prevalence estimates obtained from the two multimorbidity definitions at the provincial/territorial level	Estimation of multimorbidity prevalence
38	Frejuk et al., 2021, Canada	Prospective Cohort	≥40 353	Kidney disease	FINISHED	BP, eGFR, calcium, serum phosphorus, serum bicarbonate, Urine ACRm HBA1C	Observed significant improvements in laboratory testing in screened patients in the group compared with matched controls	Early detection of kidney disease and Screening programme

Continued on next page

Ref	Author /Year/ Country	Design	Age (Years) /N	Chronic diseases	Data source	Surveillance measures / indicators	Attribute (results)	Primary outcome
39	Garies S et al., 2021, Canada	Cohort	≥20 48,377	HTN	CPCSSN	Physical assessment, lifestyle, and risk factors	The overall crude estimates similar to the 2014–15 physical measure survey	Estimation of risk factors and crude rate
40	Hamm et al., 2021, Canada	Cohort	≥20 1.8 per 1000 population	Multi morbidity*	PHAC survey, Health insurance registries, Canada	Each province's indicators	Over 20% of registry data are available in all reporting jurisdictions, with snapshots changes in the registry	Quality improvement and management of data registry
41	Hamm et al., 2019, Canada	Cohort	≥20 0.4–1.3 per 1000 population	Asthma, COPD, DM, HTN, IHD, Stroke	CCDSS 1999–2012	Incidence per 1000 populations	Understand the changing patterns of chronic disease incidence rates	Chronic disease incidence rate
42	Hamm et al., 2022, Canada	Cohort	0–17 18 Algorithms	Juvenile DM	MCHP between 1975 and 2018	Incidence, prevalence	Control charts can be used to visualize the stability of chronic disease surveillance Incidence, 0.19 to 0.33; prevalence, 0.07 to 0.52.	Incidence and prevalence in algorithms for juvenile diabetes
43	LeBlanc AG et al., 2019, Canada	Cross sectional	All age groups 3,170,969	DM	CCDSS 2019	Incidence, Prevalence, Mortality	The CCDSS and its unique architecture provide rich data on chronic diseases in Canada	Incidence, Prevalence, and mortality
44	Lesage A et al., 2015, Canada	Cohort	All age group 903,000	Psychiatric disorders	CCDSS 2001–2002 to 2011–2012	Prevalence, Mortality, Life expectancy	Health administrative data are feasible.	The feasibility of routinely measuring the mortality gap
45	Muggah et al., 2013, Canada	Cohort	≥20 85,549	Multimorbidity*	EHR administrative data, CCHS	Prevalence, Health utility index	Self-report and administrative data useful surveillance system for many chronic diseases	Explore the administrative and survey data surveillance for chronic diseases
46	Williamson T et al., 2014, Canada	Cross sectional	≥60 1920	COPD, dementia, depression, DM, HTN, osteoarthritis, parkinsonism, and epilepsy	CPCSSN EHR	Prevalence	The CPCSSN diagnostic algorithms showed excellent sensitivity and specificity for multimorbidity*	To develop and validate case definitions and case-finding algorithms
47	Ye M et al., 2021, Canada	Cohort	≥20 52,770	Chronic diseases: Multimorbidity*	Alberta Health administrative data (October 2000–March 2018)	Incidence, Prevalence	Using administrative health data to monitor chronic diseases for large population-based prospective cohort studies is feasible	Prevalence and prevalence

Continued on next page

Ref	Author /Year/ Country	Design	Age (Years) /N	Chronic diseases	Data source	Surveillance measures / indicators	Attribute (results)	Primary outcome
48	Geda NR et al., 2021, Canada	Cross sectional	≥12 109,659	Joint pain, Asthma, COPD, Sleep apnea, Scoliosis, Fibromyalgia, Arthritis, Osteoporosis, HTN, Heart disease, Stroke, DM, Cancer, Mood disorder	2015–2016 Canadian Community Health Survey (CCHS)	Lifestyle factors and Socio-demographics	Multimorbidity prevalence = 33.3%. Higher odds with sedentary lifestyle, overweight, obesity. Risk increased with age and lower income. Smoking effect stronger in women; alcohol effect differed by immigration status.	Multimorbidity (≥2 chronic diseases) prevalence and associated lifestyle factors
49	Tomasallo CD et al., 2014, USA	Cross sectional	≥18 22,945	Asthma	UW primary care clinic data UW eHealth-PHINEX data BRFSS	Estimates the asthma prevalence	↓ Children younger than 5 (UW eHealth-PHINEX: 8.88% BRFSS: 6.16%, significantly larger percentage of females	Asthma prevalence age and sex standardized rate
50	Borunda Duque T et al., 2020, USA	Cohort	NA 280,987	DM	BRFSS January 2014–May 2019	Prevalence of DM (HbA1C) Diagnosis	Significant relationship between BRFSS and laboratory's DM prevalence estimates per counties	Disease management and prevention of NCDs
51	Klompas M et al., 2017, USA	Cohort	≥20 1,073,545	DM, Asthma, Smoking, HTN, Obesity	MDPHnet	Prevalence	MDPH net estimates at the city level showed moderate to high levels of correlation with CDC's	Estimation Prevalence rate
52	Lee DC et al., 2017, USA	Cohort	≥18 6.42 million adults	DM, HTN, Asthma	NYC CHS 2009 and 2012 SPARCS	Prevalence	Age-adjusted chronic disease surveillance NYC with emergency claims data	Age-adjusted prevalence and ED surveillance vs NYC CHS
53	Lee DC et al., 2019, USA	Cross sectional	≥25 24,141	HTN, Hyperlipidemia, DM, Cancer, Asthma, COPD/emphysema	SPARCS database 2017–2018 ED claims data, mailed health surveys	Prevalence	For select conditions, emergency claims data may be useful for tracking disease prevalence in rural areas	Prevalence estimates using ED claims data versus mailed health surveys
54	Nasuti L et al., 2022, USA	Cohort	≥40 9,130,359	Obesity, DM, high blood pressure, stroke, coronary heart disease, depression	EHR surveillance system	Estimates of disease incidence, prevalence, quality, and other health events	Data can be used in Surveillance modernization. efforts that leverage timely to address gaps	Prevalence
55	Newton-Dame et al., 2016, USA	Cohort	≥20 716,076	DM, HTN, Hyperlipidemia	NYC HNES 2013–2014, CHS–2013	Prevalence	Quality establishes documentation quality thresholds; restrict to primary care providers	Model
56	Perlman SE et al., 2017, USA	Cohort	≥20 NYC adult population	Multimorbidity*	NYC HNES, EHR 2013–2014	Prevalence	Improve data driven initiatives help achieve the full potential of EHRs for population health surveillance	Validity of indicators

Continued on next page

Ref	Author /Year/ Country	Design	Age (Years) /N	Chronic diseases	Data source	Surveillance measures / indicators	Attribute (results)	Primary outcome
57	Romo et al., 2016, USA	Cohort	≥20 9655	Depression, DM, HTN, Hypercholesterolemia	NYC HANES 2013–2014 CHS	Prevalence	Future efforts to use primary care data for chronic disease surveillance	Prevalence
58	Surbhi S et al., 2020, USA	Case study / Cohort	≥25 All adult patients ≥2 NCDs	Multimorbidity*	DWPC PBRN	Cases / prevalence	Data are powerful tools that can support real-world quality improvement in chronic disease.	Prevalence
59	Tarabichi Y et al., 2020, USA	Cohort	≥20 11.8 million	Asthma	NHIS NHAMCS CDC 2016–2017	Prevalence Asthma and ED visits	Customer collaboration and seeking to achieve nationwide Aggregate Data Program (ADP) - EHR Platforms.	Assess the feasibility of nationwide chronic disease surveillance
60	Thorpe et al., 2016, USA	Cohort	≥20 NA	HTN, Hyperlipidemia, DM	NYC HANES 2013–2014 Macroscope EHR surveillance data	Prevalence	Standardized EHR metrics have potential utility for surveillance at lower annual costs than surveys	Validation of HER findings for Chronic diseases
61	Townsend et al., 2021, USA	Mixed method	≥40 721	Colorectal cancer	EHR and Cancer registry data	Cases / prevalence	EHR data to support public health efforts and important part of chronic disease surveillance for cancer control.	Prevalence
62	Bacon et al., 2019, USA	Cohort	NA	Chronic Health Conditions	CHORDS EHRs Patient demographics	Incidence, prevalence, and management	EHR data with place-based surveillance, more timely data collection and reporting. Facilitate annual update to (CDM chronic health conditions and related procedures	Regional DDN and provides a novel method for public health surveillance
63	Scott et al., 2020, USA	Cohort and survey	≥18 2,377,939	Chronic diseases	CHORDS EHR data using PopMedNet software	Population-based survey County population (ACS) Received any care (CHAS) Received routine care (BRFSS)	Over all counties Healthcare access - 75%–80% Routine careb- 59%–63%	Compare the Record–Based Public Health Surveillance. Estimation of care seeking DDN population
64	Yang C et al., 2020, China	Cross sectional	≥21 122.4 million	chronic kidney disease (CKD)	Hospital Quality Monitoring System	Prevalence of obesity and DM diagnosis with multi morbidity	Prevalence (2007 to 2008,) 9.7% for DM and 15.5% for preDM 2013: DM at 10.9%, prevalence of HTN has increased from 12.5% (men) and 10.7% (women)	Prevalence of obesity and DM
65	Fei FR et al., 2017, China	Cohort	≥20 16.6 million	Injury related chronic diseases	Zhejiang Chronic Disease Surveillance Information and Management System	Life expectancy	Increase life expectancy after removing injury-related deaths	Life expectancy

Continued on next page

Ref	Author /Year/ Country	Design	Age (Years) /N	Chronic diseases	Data source	Surveillance measures / indicators	Attribute (results)	Primary outcome
66	Hu Y et al., 2022, China	Cross sectional	≥20 15,000 respondents in the CMHS	Mental disorders and chronic diseases	Chinese CDC) CCDRFS	Prevalence	Mood disorders and anxiety disorders with diabetes and hypertension are more serious than that of any single disease	Estimation of Prevalence of DM and HTN
67	Lai S et al., 2018, China	Cross sectional	≥18 10,568 (2003) 15,453 (2008) 48,808 (2013)	Multimorbidity	NHSS (3,4 and 5th) 2003, 2008, 2013	Prevalence	Chronic diseases are highly prevalent	Estimation Prevalence rate -
68	Ghosh A et al., 2014, Australia	Cohort	≥20 152,767	Multimorbidity *	General practice clinical systems	Case/ prevalence	Clinical data from general practice settings is both a feasible to obtain a region-specific Estimation	Prevalence of chronic disease
69	Laranjo L et al., 2016, Portugal	Cross sectional	≥50 205,068	Type 2 DM	EHR Administration data	Prevalence	EHR data shows potential to be used in public health surveillance of chronic diseases	Estimation Prevalence rate
70	Taylor et al., 2018, Australia	Cross sectional	40–69 25,621	HTN, DM, Obesity, hypercholesterolemia	PASSI, SAMSS	Prevalence Risk factors	Data indicates with positives for HBP, prevention of risk factors	Prevalence

Note: QICDSS: Quebec Integrated Chronic Disease Surveillance System; CCDSS: Canadian Chronic Disease Surveillance System; CCHS: Canadian Community Health Survey; CHMS: Canadian Health Measures Survey; NPHS: National Population Health Survey; CHD: Coronary heart disease; CVD: Cerebrovascular disease, PAD: Peripheral artery disease; CIDI-SF: Composite International Diagnostic Interview Short Form; PHAC: Public Health Agency of Canada; EMR: Electronic medical records; HRQOL: Health-related quality of life; HALE: Health-adjusted life expectancy; WBRFSS: Wisconsin Behavioral Risk Factor Surveillance System; BRFSS: Behavioral Risk Factor Surveillance System; PBRN: Population based research network; ER: Emergency room visits; SPARCS: The Statewide Planning and Research Cooperative System; NDSS: National Diabetes Surveillance System; CCDRFS: China Chronic Diseases and Risk Factors Surveillance; DWPC: DM Wellness and Prevention Coalition; MCHP: Manitoba Centre for Health Policy; CPCSSN: Canadian Primary Care Sentinel Surveillance Network; NHAMCS: National Hospital Ambulatory Medical Care Survey; UW: University of Wisconsin; CHS: Community Health Survey data; NYC HNES: New York City Health and Nutrition Examination Survey; NHSS: National Health Services Survey; PASSI: Progessi Delle Aziende Sanitane per la Salute in Italia; FINISHED: First Nations Community Based Screening to Improve Kidney Health and Prevent Dialysis; *Multimorbidity, DM, HTN, Major CVD, Unstable angina, Myocardial infarction, IHD, Heart Failure, TIA, Acute ischemic stroke, COPD, Asthma, Obstructive Sleep Apnea, Osteoarthritis, Osteoporosis, Hypothyroidism, Chronic pain, Depression; SAMSS, South Australian monitoring and surveillance system; COPD: chronic obstructive pulmonary disease; **Height, Weight, BMI, SBP, DBP, Fasting glucose, HbA1C, LDL cholesterol, HDL, cholesterol, Total cholesterol, Triglycerides, Anti-hypertensive medication, Smoking and alcohol consumption; CHORDS: Colorado Health Observation Regional Data Service; CDM: Common Data Model; DDN: Distributed data network; CHA: Colorado Health Access Survey.

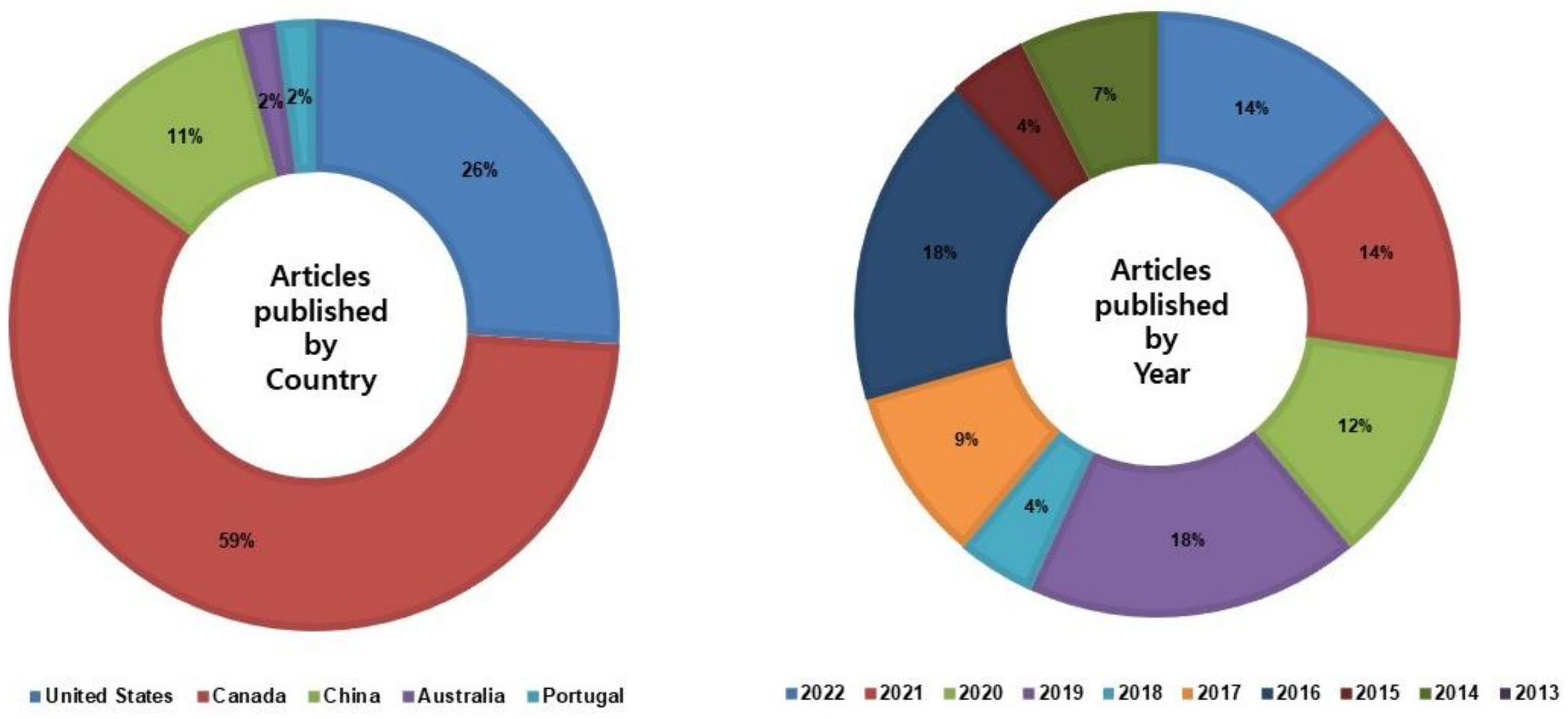

Figure S1. Articles published by country and year.

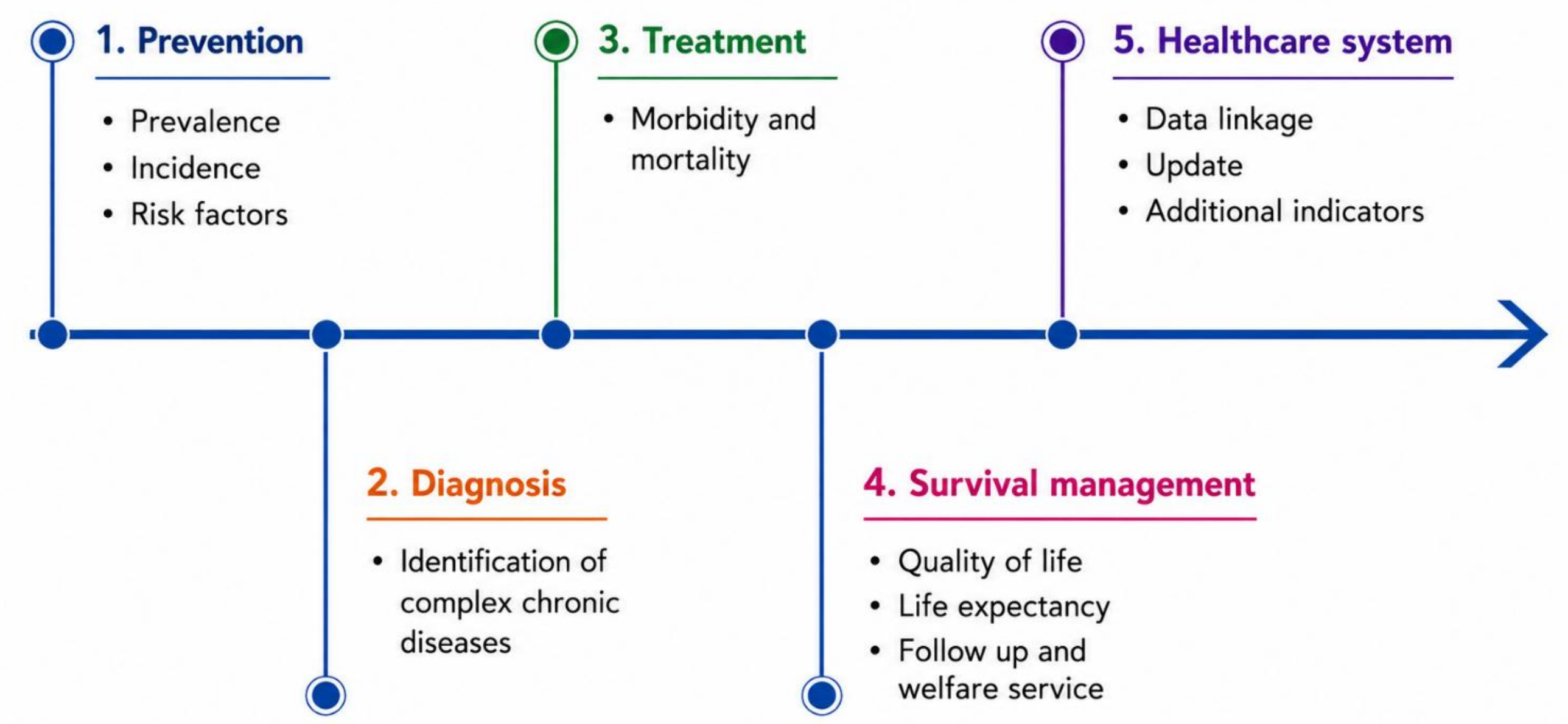

Figure S2. Chronic diseases and relevant surveillance systems were categorized by roadmap according to priority: prevalence rate, risk factors, data linkage, complex disease, and update of the existing system.

AIMS Press

© 2026 the Author(s), licensee AIMS Press. This is an open access article distributed under the terms of the Creative Commons Attribution License (http://creativecommons.org/licenses/by/4.0)
